# Identification, visualization, statistical analysis and mathematical modeling of high-feedback loops in gene regulatory networks

**DOI:** 10.1186/s12859-021-04405-z

**Published:** 2021-10-04

**Authors:** Benjamin Nordick, Tian Hong

**Affiliations:** 1 School of Genome Science and Technology, The University of Tennessee, Knoxville, TN USA; 2 Department of Biochemistry & Cellular and Molecular Biology, The University of Tennessee, Knoxville, TN USA; 3 National Institute for Mathematical and Biological Synthesis, Knoxville, TN USA

**Keywords:** Gene regulatory network, Feedback loop, Multistability, Oscillation, Epithelial-mesenchymal transition, T cell differentiation

## Abstract

**Background:**

Feedback loops in gene regulatory networks play pivotal roles in governing functional dynamics of cells. Systems approaches demonstrated characteristic dynamical features, including multistability and oscillation, of positive and negative feedback loops. Recent experiments and theories have implicated highly interconnected feedback loops (high-feedback loops) in additional nonintuitive functions, such as controlling cell differentiation rate and multistep cell lineage progression. However, it remains challenging to identify and visualize high-feedback loops in complex gene regulatory networks due to the myriad of ways in which the loops can be combined. Furthermore, it is unclear whether the high-feedback loop structures with these potential functions are widespread in biological systems. Finally, it remains challenging to understand diverse dynamical features, such as high-order multistability and oscillation, generated by individual networks containing high-feedback loops. To address these problems, we developed HiLoop, a toolkit that enables discovery, visualization, and analysis of several types of high-feedback loops in large biological networks.

**Results:**

HiLoop not only extracts high-feedback structures and visualize them in intuitive ways, but also quantifies the enrichment of overrepresented structures. Through random parameterization of mathematical models derived from target networks, HiLoop presents characteristic features of the underlying systems, including complex multistability and oscillations, in a unifying framework. Using HiLoop, we were able to analyze realistic gene regulatory networks containing dozens to hundreds of genes, and to identify many small high-feedback systems. We found more than a 100 human transcription factors involved in high-feedback loops that were not studied previously. In addition, HiLoop enabled the discovery of an enrichment of high feedback in pathways related to epithelial-mesenchymal transition.

**Conclusions:**

HiLoop makes the study of complex networks accessible without significant computational demands. It can serve as a hypothesis generator through identification and modeling of high-feedback subnetworks, or as a quantification method for motif enrichment analysis. As an example of discovery, we found that multistep cell lineage progression may be driven by either specific instances of high-feedback loops with sparse appearances, or generally enriched topologies in gene regulatory networks. We expect HiLoop’s usefulness to increase as experimental data of regulatory networks accumulate. Code is freely available for use or extension at https://github.com/BenNordick/HiLoop.

**Supplementary Information:**

The online version contains supplementary material available at 10.1186/s12859-021-04405-z.

## Background

**Fig. 1:**
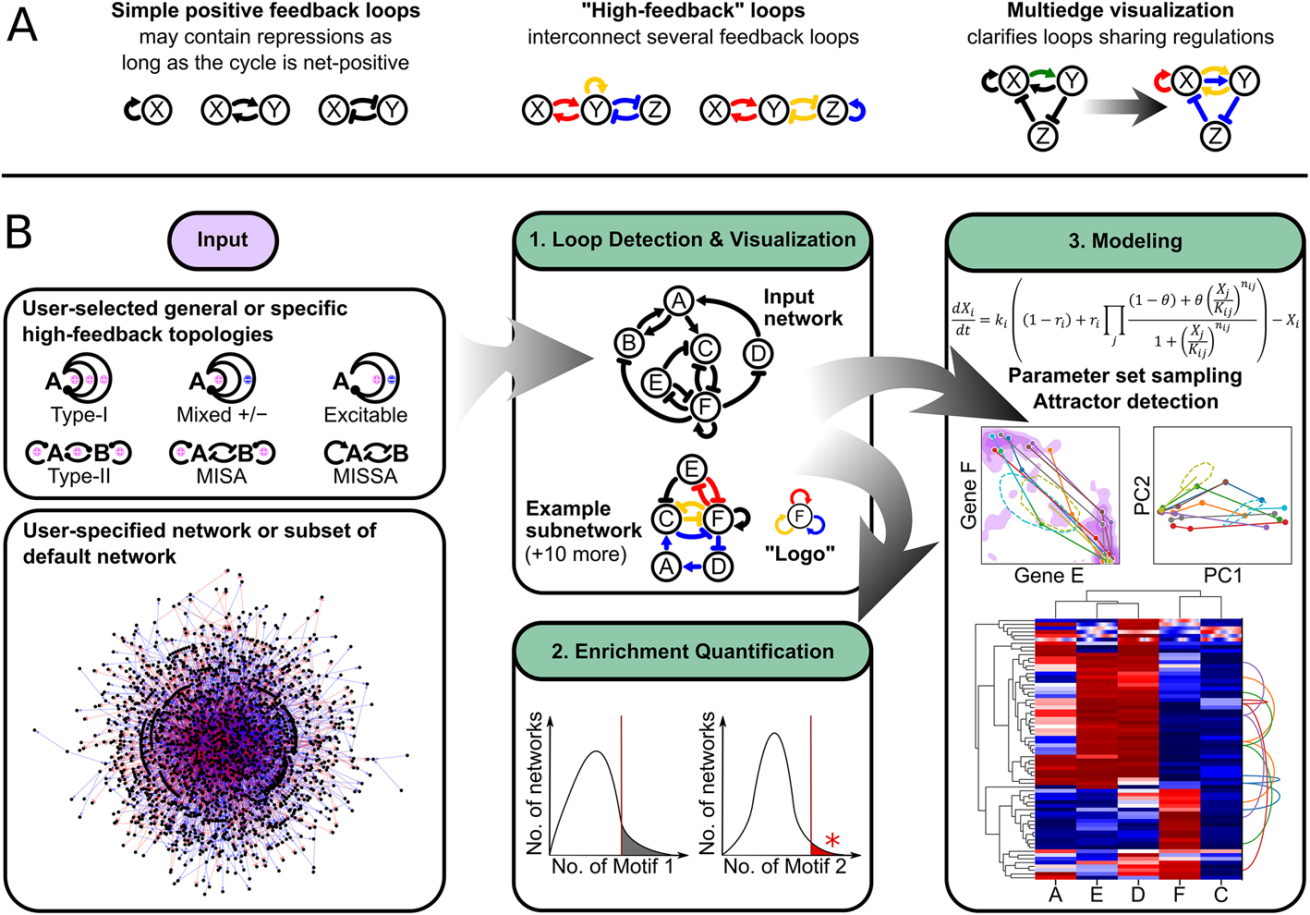
HiLoop terminology and workflow. **a** Examples of simple positive feedback loops vs. high-feedback loops. Each colored cycle is a different positive feedback loop. In high-feedback loops, one regulation may be part of multiple simple feedback loops, in which case it can be visualized as a multiedge. **b** Summary of HiLoop features and workflow. To search for high-feedback motifs, a network must be specified. This may be either a user-defined network or a subgraph of a larger existing network. Instances of various high-feedback topologies can be extracted from the input network. The upper left box shows the full list of supported cycle interconnection patterns. Cycles may contain regulations of different signs (round-head arrows) as long as the net sign matches the motif definition (+/− signs). Interconnected cycles providing high feedback are highlighted (1). To detect enrichment, the frequency of loops or specified high-feedback topologies in the input network can be compared with the frequency in randomly permuted networks (2). Detected high-feedback topologies can be modeled with sampled parameter sets and many initial conditions to visualize attractors of the different systems the network may give rise to (3). Lower-left graph depicts the TRRUST2 network with activations in blue and repressions in red; upper-middle graph depicts a subgraph of the T cell network where nodes A through F represent Eprotein, HEBAlt, Gfi1, E2A, Ikaros, and PU1 respectively

Feedback loops are well-known gene regulatory network structures responsible for dynamical behaviors important for cell fate decisions. For example, positive feedback loops generate memory of cellular decision in response to transient signals (i.e. hysteresis) [1], while negative feedback loops produce adaptive or oscillatory responses [2, 3]. More recent theoretical and experimental studies have revealed that systems of more than two interconnected feedback loops have additional roles in controlling cell dynamics, including low-rate and irreversible differentiation of adipocytes, stable intermediate cell states between epithelial and mesenchymal lineages, and stepwise lineage decision of T-cells [4–7]. Here, we define these interconnected feedback loops as high-feedback loops, a term generalized from a definition in [5] (Fig. 1a). Despite the availability of modeling and experimental studies on high-feedback structures in several specific biological systems, there is a lack of computational tools for systematically identifying and characterizing these network structures in large-scale biological networks. It is therefore unclear whether such high-feedback loops with functional dynamical properties exist widely in gene regulatory networks.

Previous studies have provided several approaches to extract network motifs from biological networks [8–11]. While these methods are useful for studying specific instances of network structures, it remains challenging to extract families of network motifs which contain diverse appearances of networks but produce unifying characteristics of dynamical systems. For example, auto-activation circuits and mutual-inhibition circuits both generate bistability, which can give rise to cellular memory when a transient signal is received. In terms of graph theory, both circuits are net-positive cycles, but they contain different numbers of nodes and edges. Numerous algorithms (surveyed in [12]) can find instances of specific subgraphs, but such methods cannot enumerate instances of high-feedback motifs, which are defined only by the signs and interconnection pattern of multiple cycles. Furthermore, due to the complexity of the high-feedback loops, visualization of these extracted network structures can be difficult: interconnected feedback loops are often combined in nonintuitive ways, and to the best of our knowledge, there does not exist a tool that can dissect the feedback elements for straightforward visual inspection. Finally, while multistability of complex gene regulatory networks has been extensively studied [13–15], it remains challenging to analyze the co-existence of multistability and other critical dynamical behaviors, such as oscillations, in the same networks.

To address these problems, we developed HiLoop, a toolkit for extraction, visualization, and analysis of high-feedback loops from large-scale biological networks. HiLoop allows identification of complex feedback loops from user-specified networks including public biological network repositories containing thousands of interactions. Each constituent feedback loop is displayed clearly so that it can be easily traced through the network. Furthermore, HiLoop quantifies the enrichment of high-feedback loops in the given networks and automatically generates parameterized mathematical models that describe characteristic dynamical systems based on the network topologies. With this toolkit, we identified potential high-feedback systems that were not previously known and made predictions about their dynamics. These systems reveal potential complex dynamical properties in various biological scenarios including cancer progression and immune cell differentiation.

## Results

### Extracting and visualizing high-feedback loops

Examples of high-feedback loops and a proposed approach to visualize them are shown in Fig. 1a. A schematic for the workflow of HiLoop is shown in Fig. 1b. HiLoop has three modules: (1) Detection and Visualization: it enumerates the appearances of a network structure in a given network, and presents them in intuitive ways; (2) Enrichment: it computes the enrichment of a network structure or its related statistics based on a background population of random networks; and (3) Modeling: it constructs dynamic models with chosen network or subnetworks, and simulates the models with random parameter sets. The simulation results are visualized with several different approaches (see later sections for details).

HiLoop offers a variety of input options. For the input network topology, it allows users to select a common motif from a list provided by HiLoop. For the input network, it allows users to define a custom network, or select genes that are used to construct a network from an existing database such as TRRUST2 [16] (Fig. 1b, left). We selected the TRRUST2 database as the suggested default for mining high-feedback loops because of its comprehensiveness. Users can expand this network by other inference methods, add custom interactions, or use a different network entirely. The user may limit the length of feedback loops for biological relevance or to ensure computational feasibility of cycle detection on large networks. The number of nodes in each extracted high-feedback subnetwork may also be limited for biological relevance. Because high-feedback motifs are defined by connections between feedback loops, HiLoop begins the high-feedback-loop detection and visualization process by finding all cycles in the input network, up to the specified length if given. It determines whether cycles overlap by testing for shared nodes. It then searches for occurrences of the requested high-feedback motifs by testing sets of overlapping cycles for the signs and interconnection pattern specified by each motif (see “Methods” for details). When a set of overlapping cycles is found to represent a motif occurrence, the subnetwork consisting of all the nodes and edges involved in the cycles is selected as an example high-feedback subnetwork if it meets the specified subnetwork size limit.

To test the functionality and performance of HiLoop, we first examined the two “types” of high-feedback loops that were shown in a previous study to facilitate stepwise lineage commitment in T cells [6]: Type-I topology containing three positive feedback loops that are connected through a common node, and Type-II topology containing a positive feedback loop between two genes, each of which is also involved in an independent positive feedback loop [5, 17, 18] (Fig. 1 and Additional file 1: Figure S1). HiLoop also includes other common high-feedback and interconnected-feedback motifs, such as mutual-inhibition-self-activation (MISA, a subset of Type-II topology) and its variants (e.g. mutual-inhibition-single-self-activation (MISSA) [19–21]), as well as paradoxical, excitable feedback that includes both positive and negative feedback loops sharing some nodes (Fig. 1) [22–25]. When counting motif instances throughout this study, we limited cycles to 5 nodes and high-feedback output topologies to 10 nodes. Five-node cycles are computationally feasible to enumerate in the several networks we examined and are known to have functional dynamics in several biological systems [26–28]. While limiting output topology size does not affect computational feasibility, it makes example subnetworks more consistent in size across different motifs.

**Fig. 2:**
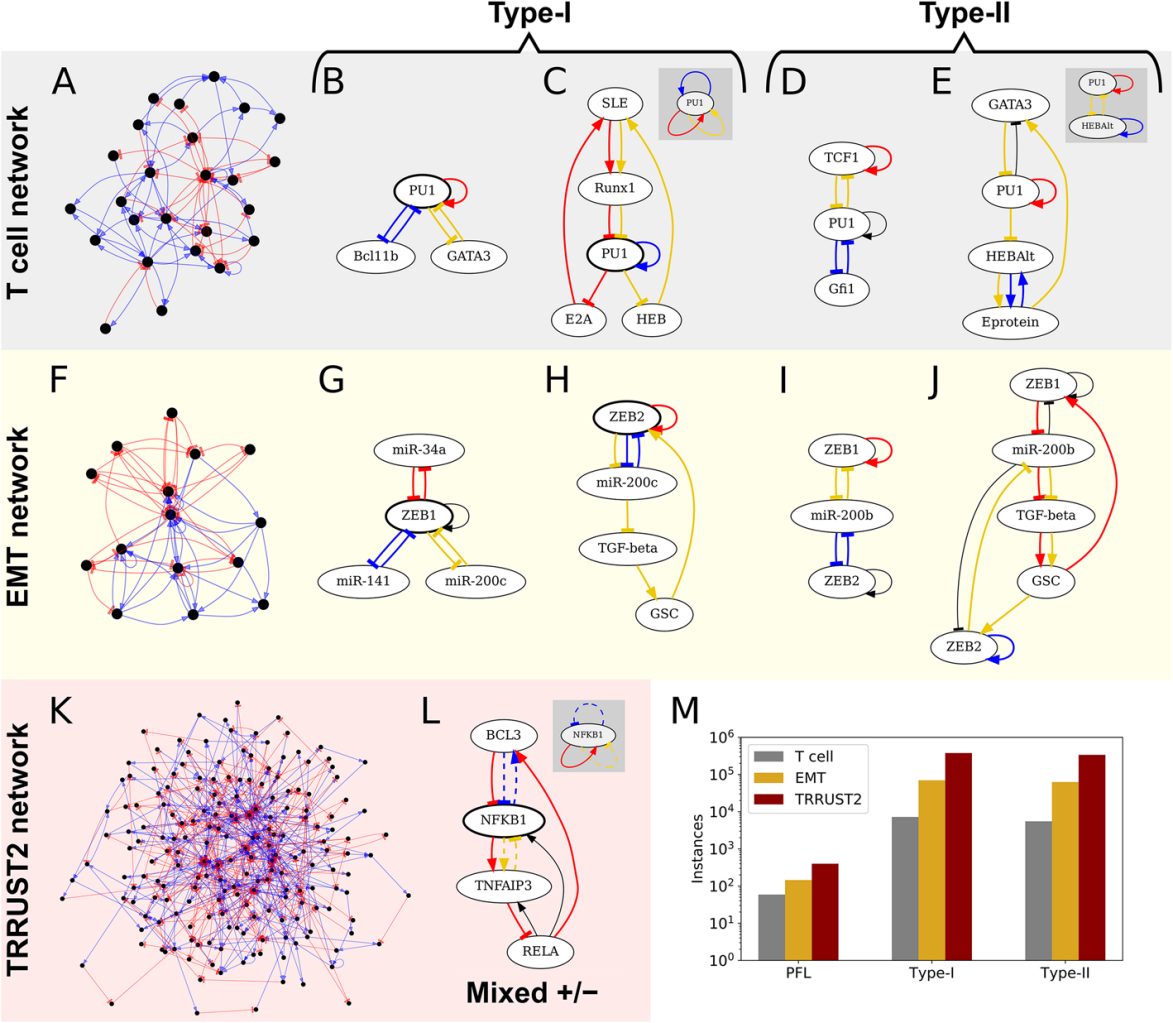
Example high-feedback topologies. Overall network structure and representative subnetworks are shown for the T cell (**a**–**e**) and EMT (**f**–**j**) networks. Left column: Overviews of the strongly connected components of the (**a**) T cell, (**f**) EMT, and (**k**) TRRUST2 networks. **b**–**e** Examples in the T cell network. **g**–**j** Examples in the EMT network. Each positive feedback loop (e.g. mutual inhibition between PU1 and Bcl11b in **b**) used by HiLoop to extract the subnetwork is highlighted in a different color. Edges with the same source and target nodes denote a single regulation involved in multiple loops (e.g. repression of PU1 by Runx1 in **c**). Additional edges induced by the set of nodes involved in the selected, colored cycles are shown in black (e.g. repression of GATA3 by PU1 in **e**). **b** A simple Type-I subnetwork of the T cell network. The node involved in all three loops is bolded. **c** A Type-I subnetwork that is more difficult to notice intuitively. Some regulations are involved in multiple feedback loops and are therefore shown multiple times. HiLoop can add a “logo” (gray panel) summarizing the motif: here, three positive feedback loops joined through PU1. **d** A Type-II subnetwork of the T-cell network. The gold-colored positive feedback loop connects the red and blue loops. **e** A Type-II subnetwork that is difficult to notice intuitively, again presented with multiedges and a logo, which for Type-II topologies shows a node from the intersection of each cycle pair. **g**, **h** Type-I subnetworks of the EMT network. **i**, **j** Type II subnetworks of the EMT network. **l** A mixed-sign high-feedback subnetwork found in the TRRUST2 network, with logo. Edges involved in negative feedback loops are dashed. **m** Positive feedback loop (PFL) and high-positive-feedback topology count in each searched network

As an example of an experimentally validated biological network, we first used a manually curated gene regulatory network for early T cell development [29]. By focusing on its strongly connected component, the largest subnetwork in which any node can be reached by following directed edges starting from any other node, we set aside nodes that are not involved in any feedback loops. This network (hereafter “the T cell network”) contains 25 nodes and 77 edges (Fig. 2a and Additional file 2: Figure S2a). HiLoop detected 7202 occurrences of Type-I topology and 5504 occurrences of Type-II topology (Additional file 3: Table S1a). As expected, many instances of these high feedback loops contain nonintuitive loop connections that are difficult to inspect visually (e.g. Fig. 1b, middle). We used HiLoop’s multigraph loop coloring to label each feedback loop clearly for intuitive inspection: regulations involved in multiple loops are drawn as multiple edges with the same source and target, making it easier to trace each loop (Figs. 1a, 2b–e). As shown in this example, there can be too many instances of a given network topology for visual inspection and modeling analyses. HiLoop therefore allows users to select manageable number of examples randomly with constraints on size and node identity.

The gene regulatory network controlling epithelial-mesenchymal transition (EMT) is another example of a biological system with highly interconnected feedback loops. Previous modeling and experimental studies have shown that interconnected positive feedback loops give rise to stable intermediate cell states between E and M states [4, 30, 31], and these intermediate states may be critical for cancer progression [32]. We obtained the strongly connected component of a previously published gene network controlling EMT [13], and used this EMT network (15 nodes and 60 edges; Fig. 2f and Additional file 2: Figure S2b) as the second example of a specific biological network. With HiLoop, we found 70,064 occurrences of Type-I topology and 62,894 occurrences of Type-II topology (Additional file 3: Table S1b). We will discuss the significance of these occurrences using statistical analysis with a background of random networks in the next section. Importantly, we not only recovered a few well-known high-feedback subnetworks that were used in previous studies (e.g. Fig. 2g/i) [4, 30, 31], but also identified subnetworks that have similar topology but are less well understood in terms of their contributions to multistate EMT (e.g. Fig. 2h/j).

Next we selected the network derived from experimentally supported human transcriptional interactions included in TRRUST2 [16]. We again focused on the strongly connected component in which every node is involved in a loop. This produced a network containing 195 nodes and 646 edges (hereafter the “TRRUST2 network”), which is larger than the T-cell and EMT networks (Fig. 2k). With HiLoop, we enumerated 378,454 instances of Type-I topology and 337,621 instances of Type-II topology in the TRRUST2 network (Additional file 3: Table S1c). Interestingly, 139 out of the 195 transcription factors in the strongly connected component were found to be involved in high-feedback topologies, suggesting potential functions of a large group of genes in governing dynamics such as stepwise lineage progression [6]. In addition, many Type-I or Type-II subnetworks include genes that are critical for fate decisions (e.g. TP53), and we will discuss the possible dynamics in a later section.

We noticed that some Type-I or Type-II subnetworks from the TRRUST2 network also included negative feedback loops. It was previously shown that combinations of positive and negative feedback loops can generate excitable systems that are critical for development [22, 25]. We therefore used HiLoop to search for pairs of interconnected positive and negative feedback loops, finding 77,426 subnetworks with this interconnection. We also searched for a generalized version of Type-I topology, in which one gene is involved in three feedback loops that do not all have the same sign (Fig. 2l). We found 2,058,336 subnetworks containing this topology, suggesting a variety of sources for excitability. Some of these subnetworks contain TP53, whose expression is known to be excitable due to interconnected feedback loops [23, 33]. Fig. 2m summarizes the instances of the feedback loops in the three biological networks discussed above.

### Enrichment analysis of network topologies and topology metrics

**Fig. 3:**
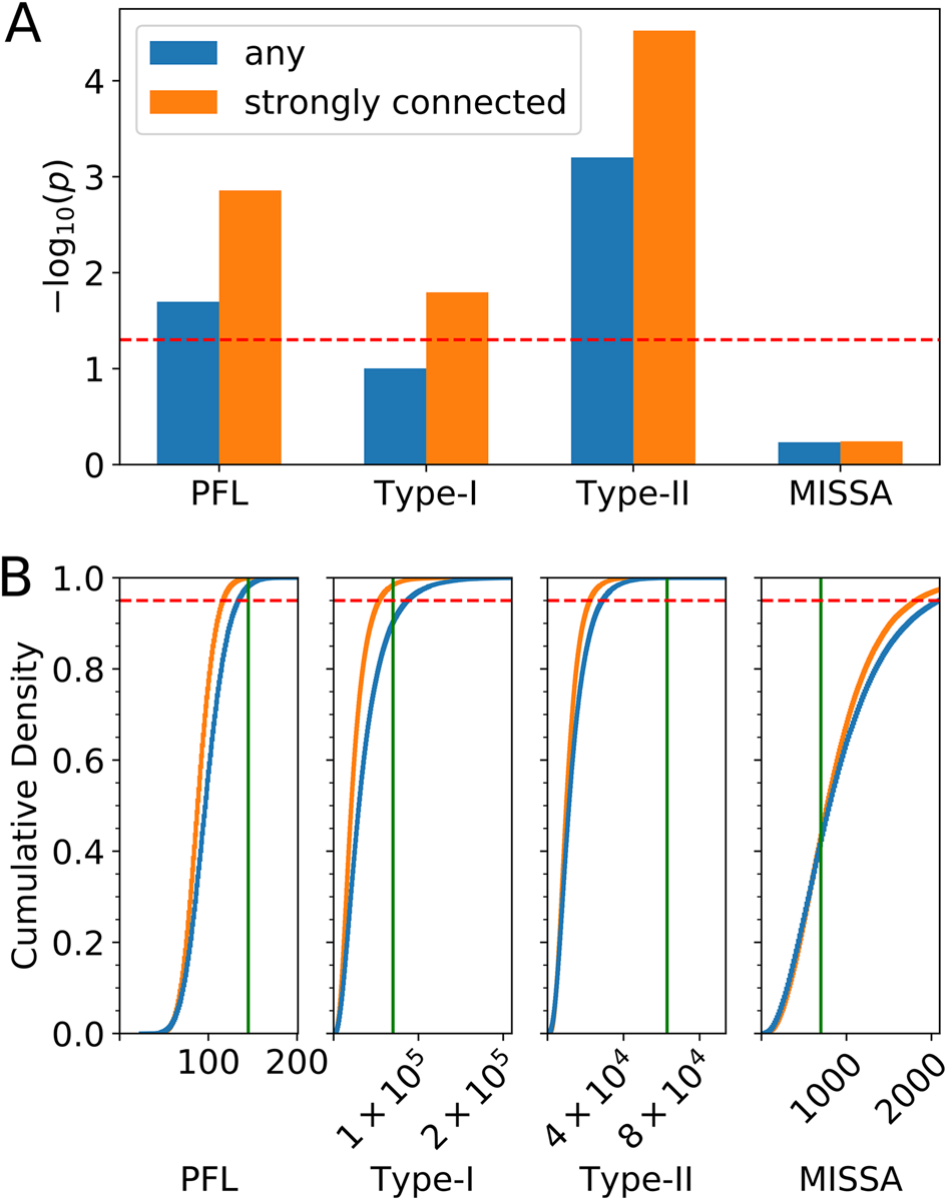
Enrichment of positive feedback and high-feedback motifs in the EMT network. **a** Empirical *p* value for enrichment of the number of positive feedback loops (PFL), Type-I motifs, Type-II motifs, or mutual-inhibition single-self-activation (MISSA) motifs. Strongly connected permutations of the strongly connected component were considered as the background for the “strongly connected” case; any permutations of the whole network were considered as the background for the “any” case. Above the dashed line, *p* < 0.05. **b** Cumulative density plots showing the proportion of permuted networks with respect to motif count cutoff. Vertical green lines show the actual EMT network’s motif counts. The empirical *p* value in **a** is one minus the cumulative density at the actual network’s count. Above the dashed line, cumulative density > 0.95, i.e. *p* < 0.05

Network topologies that are statistically enriched in a network can often enhance the robustness of certain dynamical behaviors, and the enrichment can be an indication of a positive evolutionary selection [6, 34]. HiLoop can compute empirical *p *values for the appearances of a network topology or other related quantities by generating a set of random networks with the same degree sequence, i.e. preserving the number of inbound and outbound edges for each node. Many permuted networks can be tested quickly by approximating the topology counts with a sampling-based approach (see “Methods”). With this method, we found that neither Type-I topologies, Type-II topologies, nor positive feedback loops were statistically enriched in the T cell network or the TRRUST2 network (*p* > 0.05). In contrast, Type-II topology (*p* < 0.01) and positive feedback (*p* < 0.05) are enriched in the EMT network (Fig. 3). Interestingly, a topology containing mutual inhibition between two genes and one self-activation loop (MISSA) is not significantly enriched in the same network (Fig. 3a, b). Specific instances of this topology were proposed to govern an intermediate EMT state [21, 30]. Our results raise the interesting hypothesis that the overall network properties, rather than specific modules, might be the primary mechanism underlying the observed intermediate EMT states [4, 31, 32]. In addition, the ratio of positive feedback loops to negative feedback loops is significantly high in the EMT network (*p* < 10^–5^), but not in the other two networks. These results suggest that the EMT network contains high-feedback motifs that may contribute to its characteristic dynamics (e.g. multistability) in a collective fashion, whereas similar dynamics of the T cell network or in the broader TRRUST2 network may be driven by individual genes or modules [35].

### Simulating dynamics of high-feedback loops

The potential biological functions of subnetworks obtained with HiLoop’s first two modules can be examined quantitatively through mathematical modeling. We used automated model construction and random parameterization to obtain dynamic models for time-course simulations. Details of the approach are described in “Methods”. Briefly, gene interactions are modeled with ordinary differential equations (ODEs) containing Hill functions, which are multiplied together when there is more than one regulator for a target. Instead of analyzing individual gene expression states [13], we focused on the characteristic dynamic behaviors of high-feedback loops: the coexistence of multiple attractors in individual parameter sets (i.e. multistability), and the coexistence of multistability and oscillations with individual networks.

**Fig. 4:**
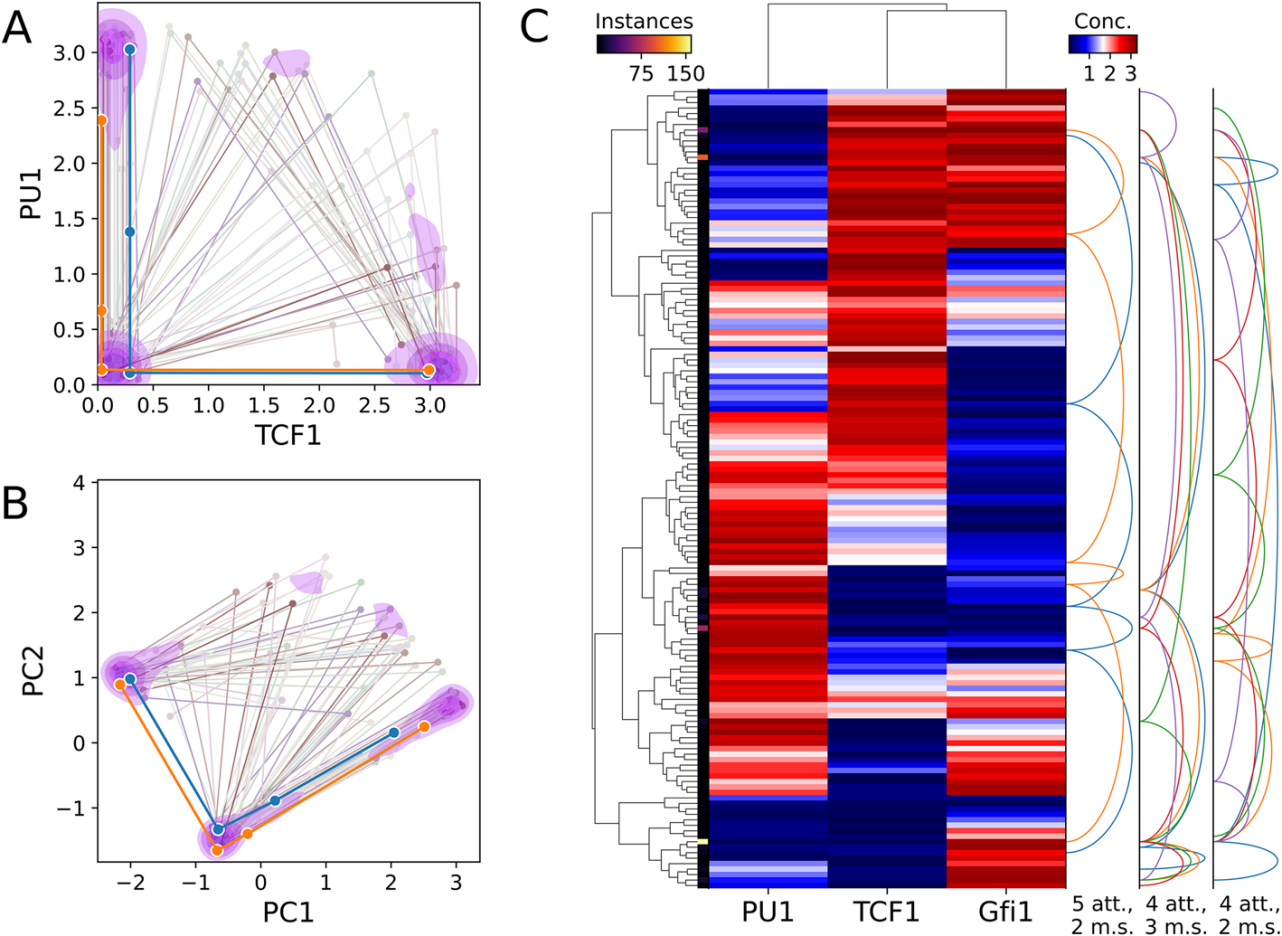
Dynamics of a three-node T cell subnetwork. Structure of the modeled network is shown in Fig. 2d. **a** Scatter-line plot of example tetrastable systems on axes of user-specified molecules’ concentrations, here TCF1 and PU1. Each line represents one system, i.e. one parameter set, that gave rise to four distinct attractors. Each point connected by a line shows the location of a distinct attractor in concentration space. The foreground thick lines represent systems where PU1 concentration never increases as TCF1 concentration increases. Contour shading reflects kernel density estimate for locations of all tetrastable systems’ attractors such that 70% of the total density is inside the pink shaded regions. **b** The same systems plotted on principal component axes. Over half of the attractors fall within one of three small clusters delineated by the second level of shading (52.5%). **c** Biclustered heatmap of attractors produced under various parameter sets tested. Attractors belonging to the same parameterization of the network are connected by a series of arcs on the right. System arcs are grouped by the number of attractors (att.) and monotonically correlated species (m.s.). Monotonic correlations of all species represent ordered attractors that may govern stepwise lineage commitment such as that of T cells [36]. Attractors within a given distance in concentration (conc.) space, here 0.2, are “folded” together and represented by one heatmap row, with brighter color along the thin left strip indicating more attractors represented by the row. The set of systems and/or system connections can be “downsampled” to reduce clutter. Here, only 10% of four-attractor or five-attractor systems discovered by the testing of one million parameter sets are shown and at most 5 systems of three selected types are connected by arcs. Gene clustering, arcs, folding, and downsampling are optional

**Fig. 5:**
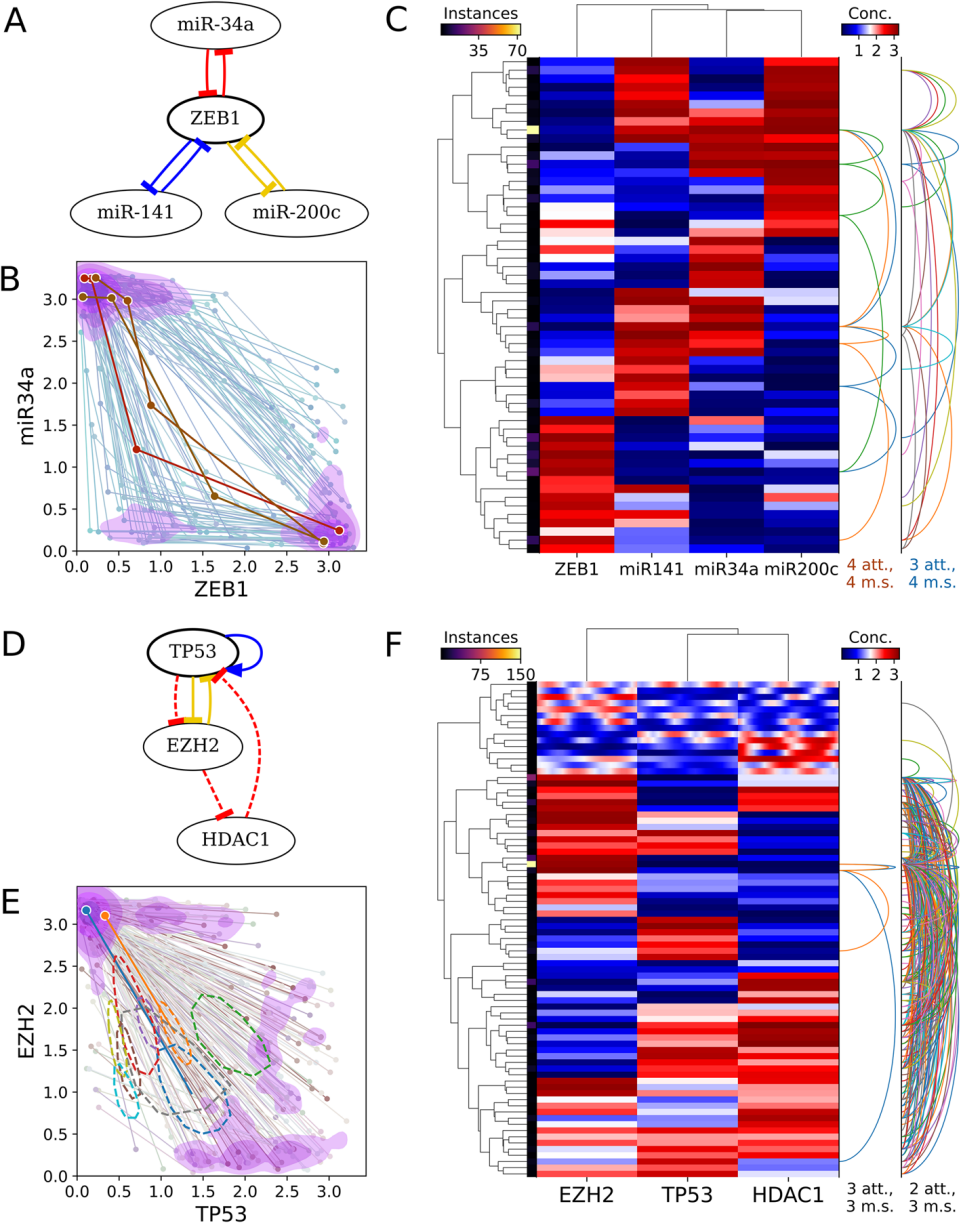
Multiattractor systems in EMT and TRRUST2 networks. **a** EMT subnetwork containing the colored edges from Fig. 2g. **b** Scatter-line plot of tristable (background, blue) and tetrastable (foreground, orange) systems found in this subnetwork, plotted on axes of ZEB1 and miR-34a concentration. Tristable systems are much more common; only 100 are shown. Each line again represents one system that gave rise to multiple distinct attractors. Each point connected by a line shows the location of a distinct attractor in concentration space. **c** Heatmap of multistable systems found in the same EMT subnetwork. Attractors within 0.5 units are folded together, only 100 tristable systems are shown, and only 10 tristable systems are traced by arc connectors. **d** A small mixed-sign high-feedback TP53-containing TRRUST2 subnetwork capable of generating oscillatory attractors. Dashed cycle, negative feedback loop. **e** Scatter-line plot of bistable or oscillation-containing systems found in the subnetwork shown in **c**. Oscillatory attractors are shown as a thick dashed line tracing the orbit. Most oscillators are the only attractor in their system. **f** Biclustered heatmap of the same systems. Oscillatory attractors are shown as gradients instead of solid-colored cells

We modeled 100 representative Type-I or Type-II high-feedback subnetworks of at most 5 nodes obtained from the T cell network. We found that 72 subnetworks were able to produce four coexisting attractors within a parameter set. Moreover, 59 modeled subnetworks produced four-attractor systems in which all attractors were monotonically ordered in the expression space of every involved gene (Fig. 4). These properties may be critical for the multistep lineage progression of developing T cells in the thymus [36]. We obtained similar results from the EMT network in terms of the ability of high feedback to produce ordered attractors (Fig. 5a–c).

HiLoop visualizes multiple attractors in the state space of specific genes or axes of reduced dimensions obtained from principal component analysis (Fig. 4a, b). In addition, it provides biclustering analysis of genes and states obtained from random parameter sets (Fig. 4c). Most importantly, the information of multistability is retained in both visualization approaches (lines in the scatter plot and arc connectors on the right of the heatmap), a crucial feature allowing users to test hypotheses about multistability with specific gene expression patterns. For example, ordered tetrastable systems of the network examined in Fig. 4 are unlikely to contain any attractor of intermediate TCF1 concentration, while they likely have an attractor of intermediate PU1 concentration and low TCF1 concentration as a step between high-PU1 and low-PU1 states.

As expected, we found that TRRUST2-derived subnetworks containing Type-I and Type-II topologies were able to generate more than two attractors with individual parameter sets. Because several genes in these networks are known factors controlling cell cycle progression and differentiation (e.g. JUN, TP53, MYC, FOXP3; see Additional file 3: Table S1c), our results predict the existence of high degrees of multistability during cell cycle progression or lineage commitment arising from these interconnected feedback loops. More interestingly, the coexistence of both positive and negative feedbacks in those TRRUST2-derived high-feedback-loop subnetworks led to the hypothesis that individual networks may generate both multistability and steady state oscillations. Indeed, the modeling module of HiLoop captured these distinct dynamical features (Fig. 5d–f). Our results with a network containing TP53, which can produce bistable systems or oscillations depending on the parameter set, are consistent with a previous model which showed the possibility of coexisting oscillatory and bistable systems in adjacent parameter regions during DNA-damage responses [37]. Notably, the visualization methods in HiLoop allow the presentation of oscillation and multistability in the same scatterplots and cluster-heatmaps. This visualization method allows us to inspect specific findings regarding the dynamical systems. For example, coexistence of an oscillatory attractor and a point attractor with the same parameter values occurred very rarely in the sampled sets (Fig. 5f, top section of the right arc plot).

## Discussion

Numerous theoretical studies have shown the importance of interconnected feedback loops [6, 17, 38–40], but automated extraction of those complex loop structures from realistic and large-scale biological circuits has been challenging. This prevents deeper understanding of these networks in a wide range of biological systems. While several existing tools can be used to examine robustness of feedback loops [41, 42], they do not have the capacity of extracting and analyzing interconnected or high-feedback loops. The novel analytical capacities of HiLoop enable biologists to test hypotheses involving complex feedback loops in the context of large-scale networks.

Because the main strongly connected component (SCC) of a network, by definition, does not contain nodes that do not participate in any feedback loop, enumeration of feedback-rich motifs proceeds equivalently on the SCC and the whole original network. Enrichment quantification, however, can be sensitive to whether the whole network or its SCC is used as a base for permutation. Starting with the SCC removes edges that could be rearranged to form additional feedback loops and high-feedback topologies, so it can give an impression of stronger enrichment. We therefore recommend the use of the whole original network of interest for a conservative, reliable determination of motif enrichment.

Existing packages, such as GeneEx and RACIPE, are useful for examining the robustness of gene regulatory networks in terms of gene expression patterns [13, 15]. However, functions for network motif analysis are limited in these tools. In addition to motif searching and statistical calculations, HiLoop provides several additional useful features, including a default comprehensive transcriptional network, and visualization of both multistability and oscillatory gene expression patterns. To the best of our knowledge, these features are not available in existing tools. While HiLoop currently only supports the visualization of steady state behaviors of temporal models, we expect that our visualization method can be extended to facilitate the analysis of diverse transient dynamics and spatial (e.g. Turing) patterns generated from individual spatiotemporal models [28, 43, 44].

For the input network, HiLoop supports both user-defined networks and a built-in systems-wide transcriptional network from TRRUST2. A user-defined network might be obtained with network inference algorithms that produce signed directed graphs from omics data [45, 46]. It should be noted that some existing network inference methods may underestimate the number of feedback loops and mutual interactions [47]. For this reason, network inference is not implemented in the current version of HiLoop, but will be a direction of future development.

## Conclusions

We systematically enumerated high-feedback topologies in several realistic biological networks, finding that over one hundred human transcription factors have the topological potential to contribute to multistability. We determined that a network of genes controlling epithelial-mesenchymal transition is significantly enriched in positive feedback and in a specific motif of interconnected positive feedback loops. We demonstrated the capacity of automatically constructed ODE models to reproduce experimental observations of sophisticated dynamics such as a switch between a steady state and an oscillation.

Our software toolbox HiLoop facilitates all these methods and allows visualizing their results. As the development of comprehensive gene regulatory network databases and inference methods continues, the analytical capabilities of HiLoop can be further utilized by diverse biologists interested in analyzing complex gene regulatory networks in a wide range of biological systems.

## Methods

### TRRUST2 data import

The full table of human transcription regulation relationships was downloaded from the TRRUST version 2 web page. Distinct genes, whether source or target, were added as nodes to a directed graph. Interactions were added as edges. When conflicting signs were proposed for one interaction, the sign associated with more citations was selected. Interactions of “unknown” sign or for which activation and repression were supported by the same number of citations were discarded.

### Motif enumeration

Systematically counting instances of a high-feedback motif requires finding every subnetwork, i.e. set of edges, within the given network that can be produced by selecting a small set of cycles according to the motif definition. Since high-feedback motifs are defined in terms of cycles and cycles are comprised of edges, a set of cycles specifies exactly one topology within the network. The reverse, however, is not true; because cycles can overlap, multiple different sets of cycles could specify the same set of edges. Directly screening all sets of edges is infeasible for realistic networks, so sets of cycles must be enumerated in such a way that each motif-conforming set of edges is counted only once. Maintaining a collection of every topology already counted is also infeasible for large networks, but finding additional cycles that could be used in an alternate representation of a potentially new topology is feasible, as discussed below. The ability to compare selected cycles to additional cycles makes it possible to recognize a “canonical” set of cycles for each topology without holding previously counted topologies in memory. This comparison requires that all cycles can be placed in some total order. For example, cycles could be compared in lexicographic, “dictionary” order by the names of the nodes they visit. In practice, each cycle is associated with a unique numeric identifier such as a memory address for efficient comparison.

**Fig. 6:**
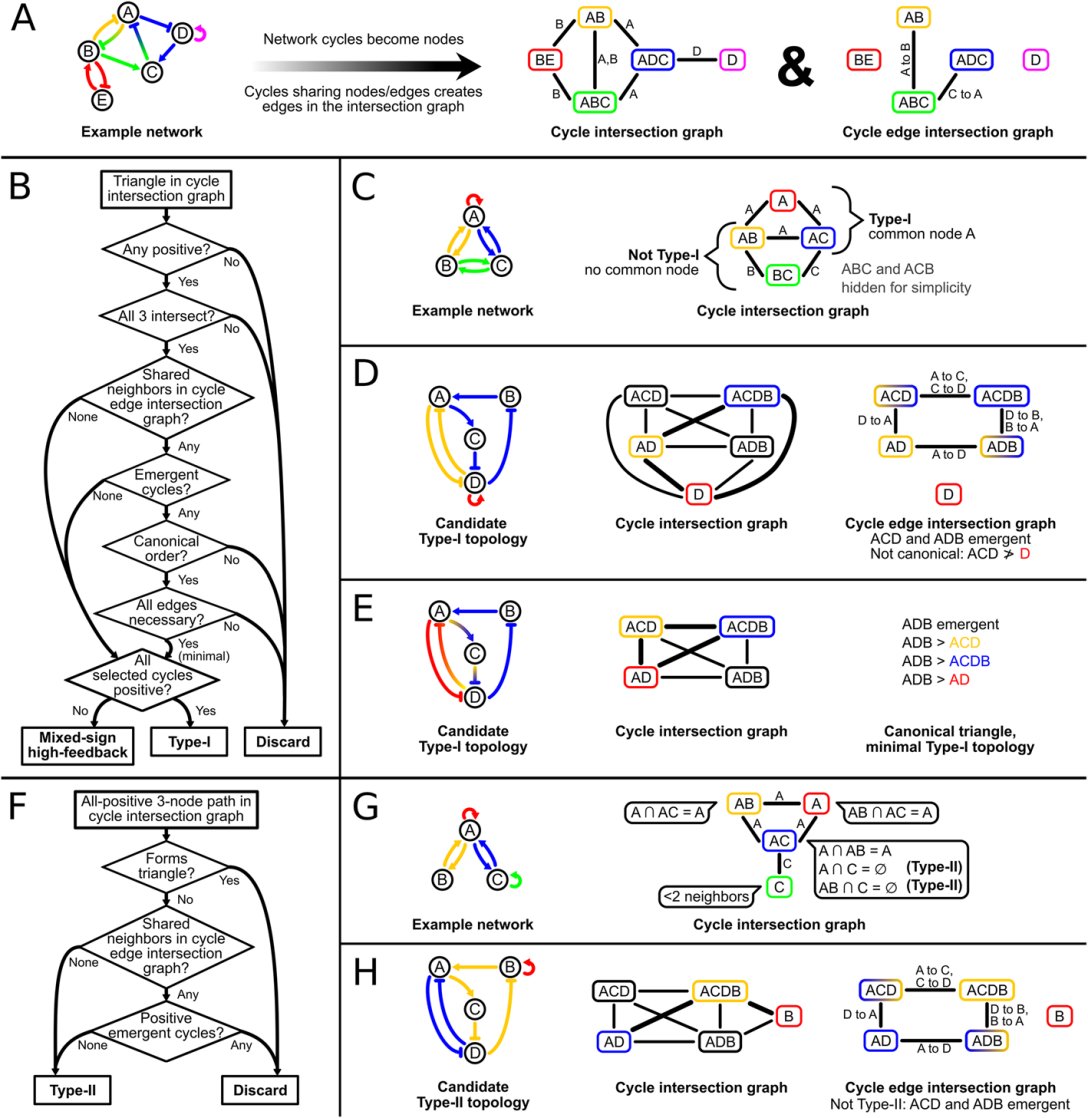
Data structures, procedures, and challenges in high-feedback topology enumeration. **a** Cycle intersection and cycle edge intersection graphs for an example network. Cycles are named for their nodes in the order visited. **b** Summary of Type-I and mixed-sign high-feedback identification. **c** Identification of candidate Type-I topologies by examining triangles in the cycle intersection graph. **d** Elimination of duplicate or nonminimal Type-I topologies by detecting emergent cycles. Selected cycles ACDB and AD both have ACD and ADB as neighbors in the cycle edge intersection graph. All edges in ACD or ADB are in the union of ACDB and AD, so they are emergent cycles (gradients in cycle edge intersection graph). If cycles are ordered lexicographically, the emergent cycle ACD is before selected cycle D, so the selected triangle (thick lines) cannot be canonical. Even if the order was correct, removing the D self-loop leaves at least 3 interconnected cycles, so this topology is not minimal. Example shown is based on a subnetwork of a core T cell regulatory network [6]. **e** The previous network without the self-loop. Gradients indicate edges involved in multiple selected cycles. This topology is minimal because removing any edge leaves fewer than 3 cycles. If cycles are ordered lexicographically, the selected triangle is the canonical representation of this topology because the only emergent cycle ADB is ordered after all the selected triangle’s cycles. **f** Summary of Type-II identification. **g** Identification of candidate Type-II topologies by examining pairs of neighbors in the cycle intersection graph. The callout on each potential bridge cycle lists all possible pairs of neighbor cycles and whether they are independent. **h** Elimination of nonminimal Type-I topologies from Type-II candidates. Cycle AD combined with bridge cycle ACDB creates emergent cycles ACD and ADB, so this topology is not minimal. Example shown is a different subnetwork of the network used in **d**

Networks are represented and manipulated as directed graphs using the NetworkX Python package [48]. Cycles of limited length are first enumerated by the algorithm of Liu and Wang [49]. For each cycle, the net sign, presence of any repression edges, and set of edges—each represented as a pair of node IDs—is cached in an object representing the cycle. A unique numeric identifier of this object enables total ordering of cycles. A simple undirected “cycle intersection” graph is created such that each cycle in the original network is represented by a node and each edge connects a pair of cycles that share at least one node [50] (Fig. 6a). Similarly, a “cycle edge intersection” graph is created such that each edge connects a pair of cycles that share at least one edge. In both derived graphs, each node is tagged with the set of original node IDs involved in the cycle and each edge is tagged with the set of shared items.

The process of detecting Type-I topologies, which consist of three positive feedback loops sharing at least one node, and mixed-sign high-feedback networks, which consist of three feedback loops that are neither all positive nor all negative, is summarized in Fig. 6b. Triangles of the cycle intersection graph are enumerated to search for interconnected triplets of cycles (Fig. 6c). Triangles representing triplets of negative cycles are discarded because both motifs involve at least one positive feedback loop. Triangles in which the intersection of the three edges’ tag sets is empty, i.e. there are no nodes involved in all three cycles, or the union of the cycles’ nodes exceeds the maximum motif size, if specified, are discarded. Because the combination of two or more cycles may produce new cycles, the subnetwork specified by the three cycles of the triangle may contain additional, “emergent” cycles, which could contribute to alternate representations of the same subnetwork. Emergent cycles share edges with already selected cycles, so cycles neighboring at least two of the triangle’s three cycles in the cycle edge intersection graph are enumerated. Such a candidate is an emergent cycle if each of its edges is also in at least one of the three selected cycles (Fig. 6d). Once all selected and emergent cycles are known, the triangle’s canonicality is determined by the order of the emergent cycles relative to the selected cycles: subject to the motif definition, the cycles ordered as early as possible must be in the triangle (Fig. 6d, e). Because Type-I topologies are defined by only positive feedback loops, emergent negative cycles are allowed to precede the positive cycles in the triangle. Because mixed-sign high-feedback motifs require at least one cycle of each sign, emergent cycles may precede the selected cycle of the unique sign if they are of the opposite sign; they could not “replace” that selected cycle without abolishing the mixed-sign nature of the topology. Even after noncanonical triangles are discarded to avoid double counting, the presence of emergent cycles allows for nonminimal topologies, which have more edges than necessary to meet the high-feedback motif definition. To count only minimal topologies for consistency with previous work [6], the removal of each edge is tested to determine whether the reduced topology still meets the motif definition. If an edge can be removed without abolishing the motif, the triangle is discarded as nonminimal (Fig. 6d). Remaining triangles represent Type-I or mixed-sign high-feedback topologies depending on the signs of their constituent cycles.

The process of detecting Type-II topologies, which consist of two non-overlapping positive feedback loops bridged by a third positive feedback loop, is summarized in Fig. 6f. Pairs of positive neighbors of each positive cycle in the cycle intersection graph are enumerated, forming paths of three nodes in which the middle node represents the “bridge” cycle (Fig. 6g). Paths in which the union of the three nodes’ tag sets is larger than the maximum motif size are discarded. To avoid counting nonminimal Type-I topologies as Type-II, paths are discarded if they form a triangle in the cycle intersection graph with the bridge cycle or if an additional positive cycle emerges when the bridge cycle’s edges are combined with those of either outer cycle (Fig. 6h). Remaining paths represent Type-II topologies. A Type-II topology is also considered a MISA topology if the two paths of the bridge cycle that connect the outer cycles each contain an odd number of repressions, i.e. if there is net-inhibition in both directions via the bridge (Additional file 1: Figure S1c).

To detect excitable or MISSA motifs, edges in the cycle intersection graph, i.e. interconnected pairs of cycles, are examined. Cycle pairs whose union is larger than the maximum motif size are discarded. Edges that connect one positive and one negative feedback loop constitute excitable topologies (Additional file 1: Figure S1e). Edges that connect two positive feedback loops constitute MISSA topologies if one loop involves repressions, providing mutual inhibition between a shared node and a node it represses in this loop, and the other loop involves only activating interactions (Additional file 1: Figure S1f-g). Alternatively, a more restrictive “mini-MISSA” definition may be used, which requires the activation-only positive feedback loop to be a direct self-loop.

On 64-bit Ubuntu 20.04 in Hyper-V on a computer with an Intel Core i7-3770 processor, counting Type-I, Type-II, MISA, and MISSA topologies in the TRRUST2 network, limited to 5 nodes per cycle and 10 nodes per topology, took 54 s of wall clock time. Counting negative-feedback-containing motifs as well, which increases the number of cycles and especially triangles that must be considered, took 745 s (12 m 25 s).

### Enrichment analysis

To determine the enrichment of a motif in a network, the motif’s frequency in the original network is compared to its frequency in permuted versions of the network. The space of directed graphs with the same degree sequence is used for a realistic background [51]. Prior to edge rearrangement, edge signs are randomized preserving number of repressions. During edge rearrangement, each permutation step first attempts a double-edge swap between two randomly selected edges and, if a new edge would conflict with an existing edge, instead randomly selects a predecessor of the first edge’s source and attempts to reverse a directed triangle consisting of the predecessor, first edge’s source, and first edge’s destination if such a triangle exists. The number of steps used in generating each permuted network is randomly selected between one and three times the number of edges in the network. MicroRNA species can only repress, so after edge rearrangement, each activation out-edge from a miRNA node is sign-swapped with a randomly selected repression edge elsewhere in the network to preserve the number of repressions. If only the space of strongly connected permutations is being tested, non-strongly-connected permutations are not used for motif detection, but still serve as the base for the next round of permutation to ensure that the space of graphs can be fully explored.

Because it is costly to compute the exact number of occurrences of each topology in all background (permuted) networks, we use a sampling approach to approximate the topology counts in each permuted network. Cycles of limited length are enumerated in each network [49]. Each network’s collection of cycles is sampled many times. Each sample is a cycle triplet: three cycles selected randomly, regardless of feedback loop positivity. The cycle pair consisting of the first two selected cycles is first examined. If the union of the pair contains more than the maximum number of nodes, the sample is discarded. Pairs with non-empty intersection in which exactly one cycle is net-positive constitute excitable samples. Pairs with non-empty intersection in which both cycles are positive but exactly one contains any repressions constitute MISSA samples. Once two-cycle motifs have been considered, cycle triplets whose union contains more than the maximum number of nodes are not examined further. Triplets whose intersection is non-empty constitute Type-I samples if all three feedback loops are positive or else mixed-sign high-feedback samples if at least one is positive. Likewise, triplets of positive feedback loops in which two cycles have an empty intersection with each other but nonempty intersections with the third cycle constitute Type-II samples. For each motif, the actual number of instances $a$ in a permuted network can be estimated by

1 $$a \approx \frac{ks}{N}\left( {\begin{array}{*{20}c} c \\ m \\ \end{array} } \right) .$$

In this expression, *s* is the number of samples that contain the motif, *N* is the number of triplets sampled, *c* is the number of cycles in the network, and *m* is the number of cycles that must interact to comprise an instance of the motif. The constant of proportionality *k* differs by base network and motif, but because the relationship is monotonic and nearly linear in $s$ (Additional file 4: Figure S3), this expression can be used without *k* to compare permutations to the base network. Positive feedback loop count and ratio of positive feedback loops to cycles are computed exactly. The empirical *p* value for enrichment of a motif is the proportion of permuted networks with at least as many instances of the motif as the base network. Several sampling runs of the base network may be averaged to reduce noise in the estimate of its motif count.

Enrichment of the EMT network was computed by sampling 1000 cycle triplets from each of 100,000 permutations, averaging 100 sampling runs of the base network, limiting cycle length to 5 nodes, and limiting motif instances to 10 nodes. For display in Fig. 3, motif count estimates were scaled by a constant factor so that the base network’s average count matched the exact count of the same network as shown in Fig. 2.

### Example motif selection

Pairs and triplets are sampled from the collection of length-limited cycles as described above. Cycle tuples that do not conform to a previously described cycle interconnection motif are discarded. The union of nodes in the selected cycles is used to extract the induced subgraph. Optionally, some edges in the induced subgraph that are not part of any of the original three cycles may be randomly eliminated. Subnetworks with the same edge set as an already selected subnetwork or that violate the input constraints on motif size, edge count, or number of nodes shared with an already selected subnetwork are discarded. When a subnetwork is selected as an example for visualization, each of the cycles used to induce it is highlighted a different color. Edges that must be colored twice, i.e. that appear in multiple such cycles, are duplicated so that each cycle can be easily visualized. Graph images are rendered using the PyGraphviz binding to Graphviz using the “dot” engine for layout [52]. A GraphML file representing each network may also be saved for modeling or further analysis. The cycle tuple sampling process continues until the requested number of examples of each motif have been found.

Constraints on subnetwork size and/or node identity can be specified to select a manageable number of example subnetworks for visualization or modeling. To test dynamics of the T cell and EMT networks, 50 Type-I and 50 Type-II examples of each were selected with a maximum cycle length of 4 nodes, maximum subnetwork size of 5 nodes, and elimination of additional induced edges enabled. For display of example networks in high-quality figures, minor Graphviz rendering artifacts that appear at high resolution were manually corrected.

### Mathematical models

To model the dynamics of a network, its NetworkX graph is converted to Antimony models representing a system of ordinary differential equations (ODEs) [53]. Each node in the network becomes one ODE with a Hill term for each in-edge [6, 40, 54]. Each ODE is of the form

2 $$\frac{{dX_{i} }}{dt} = g_{i} \left( {k_{i} \left( {\left( {1 - r_{i} } \right) + r_{i} \mathop \prod \limits_{j} \frac{{\left( {1 - \theta_{ij} } \right) + \theta_{ij} \left( {\frac{{X_{j} }}{{K_{ij} }}} \right)^{{n_{ij} }} }}{{1 + \left( {\frac{{X_{j} }}{{K_{ij} }}} \right)^{{n_{ij} }} }}} \right) - X_{i} } \right) .$$

Here, $X_{i}$ is the concentration of the species (i.e. gene product) $i$, $g_{i}$ is its timescale, $k_{i}$ is its maximum synthesis rate, and $r_{i}$ is the sensitivity of the synthesis rate to regulation. In each term of the Hill product, $\theta_{ij}$ is 1 for activation edges or 0 for repression edges, $X_{j}$ is the concentration of the regulator, $K_{ij}$ is the threshold of the regulation, and $n_{ij}$ is the cooperativity. The generalized form of Hill function allows us to model gene regulations in a standardized manner. Capturing dynamics of posttranscriptional or posttranslational regulations may require more detailed descriptions, such as mass action kinetics [55, 56]. The network connectivity revealed by the current modeling framework of HiLoop is therefore considered sufficient, rather than necessary, for the target dynamics.

For each parameter set, parameter values are randomized within ranges specified in Table 1 and used by previous work [6] for efficiently finding multistable parameterizations. In particular, since the threshold parameter $K_{ij}$ is sampled in a wide range, which allows sufficient flexibility of each regulation, parameters $r_{i}$ and $k_{i}$ are sampled in relatively narrow ranges (Table 1) by default. These ranges can be straightforwardly adjusted if desired. To improve simulation efficiency, all timescales and therefore degradation rates are fixed at 1 unless $g$ sampling is explicitly enabled, which can be critical for capturing certain temporal dynamics such as oscillation.

**Table 1:** Randomized parameter ranges for multistability detection

Parameter	Description	Minimum	Maximum
$g_{i}$	Timescale of $i$ (log-uniform distribution, fixed at 1 by default)	0.1	10
$k_{i}$	Maximum synthesis rate of $i$	3.0	3.3
$r_{i}$	Proportion of the synthesis rate of $i$ reliant on regulation	0.90	0.99
$K_{ij}$	Threshold of the regulation of $i$ by $j$	0.05	4.50
$n_{ij}$	Cooperativity of the regulation of $i$ by $j$ (integer)	1	6

All parameters are drawn from a continuous uniform distribution unless otherwise specified

The system is simulated numerically using Tellurium [53] for all combinations of initial concentrations, each evenly spaced between 0 and 3.3. The endpoint of each simulation is classified as a point attractor if no concentrations vary in the last 10 time units, an oscillatory attractor if the endpoint concentration vector appears multiple times over the time course while all concentration ranges are consistent over the last half of the simulation, or unstable otherwise. Point attractors more than 0.5 units apart in gene concentration space are considered distinct. Oscillatory attractors are distinguished by their extrema and Fourier transform peaks as computed with the NumPy [57] and SciPy libraries [58]. Unstable endpoints are discarded. Each parameter set that gave rise to a system with the requested multi-attractor or oscillatory characteristics is written to a JavaScript Object Notation (JSON) file for further analysis or visualization of the systems.

Two species are considered “monotonically correlated” in a system if sorting the list of attractors by the concentration of one species causes it to also be sorted by the concentration of the other, regardless of direction. A system’s number of “monotonically correlated species” is the size of the largest set of species that are all monotonically correlated with each other.

Examples from the T-cell network were tested for multistability by simulating 4 initial concentrations per node in each of 1,000,000 parameter sets per subnetwork for 100 time units. Only systems containing exclusively point attractors were considered.

### Dynamics visualization

Attractor data is loaded from a JSON report into a matrix with one row per attractor and one column per species [57]. When expressed as points, oscillatory attractors are positioned at the mean of the orbit based on the concentration of each gene product. For display in 2D scatter-line plots, the matrix is reduced to two user-selected columns or to the first two principal components as computed by scikit-learn [59]. The Gaussian kernel density estimate for contour steps is computed using scikit-learn with a bandwidth of 0.1. Heatmap cores and dendrograms are rendered with the seaborn library [60]. Linkage is computed with SciPy using the average method and Euclidean metric [58] on an expanded matrix with an additional column for the average speed of change of each species’ concentration. To optionally link all oscillatory attractors together, an additional column contains 0 for point attractors and a user-specified scalar—2 in Fig. 5f—for oscillatory attractors. Additional visual elements and other plots are rendered with the matplotlib library [61].

## Supplementary Information

**Additional file 1. Figure S1:** Examples of supported motifs, with logo, found in the TRRUST2 network. **a** A Type-I topology, consisting of three fused positive feedback loops. **b** A Type-II topology, consisting of two independent positive feedback loops bridged by a third net-positive loop. The bridge loop may or may not contribute mutual inhibition. **c** A mutual inhibition self-activation (MISA) topology, i.e. a Type-II topology with mutual inhibition between members of the two independent positive feedback loops. **d** Mixed-sign high-feedback topologies, consisting of three fused feedback loops, at least one of which is positive and at least one of which is negative. Dashed lines in the network diagram and logo indicate which cycles are negative. **e** An excitable topology, consisting of a positive feedback loop fused to a negative feedback loop. **f** A mutual-inhibition single-self-activation (MISSA) topology, in which a pure-activation feedback loop is fused to another positive feedback loop that does contain repressions. The self-activation may or may not be a self-loop. **g** A “miniature” MISSA topology in which the self-activation is a self-loop.

**Additional file 2. Figure S2:** Structures of the (**a**) T cell and (**b**) EMT network. Nodes and edges outside the strongly connected component are gray.

**Additional file 3. Table S1:** Number of high-feedback topologies containing each node in the (**a**) T cell, (**b**) EMT, and (**c**) TRRUST2 strongly connected components, limiting cycles to 5 nodes and topologies to 10 nodes.

**Additional file 4. Figure S3:** Cycle tuple sampling well approximates motif counting for purposes of enrichment detection. Scatterplots show motif instances as determined by exhaustive counting vs. the cycle tuple sampling approximation for 500 permutations of the T cell, EMT, and TRRUST2 networks. The slope k is the constant of proportionality from Equation 1. “SCC” rows tested only strongly connected permutations of the network’s strongly connected component; other rows tested any permutations of the full network. A red dot represents the original, unpermuted network (far outside the permutation range for EMT Type-II). Empirical p_count_ is the proportion of permutations with an exhaustive count greater than the original network’s; empirical p_sample_ is the proportion of permutations with a sampling-based estimate greater than the original network’s.

**Additional file 5. Repository snapshot (ZIP):** Archive of the HiLoop repository, including code and network structures.

## Data Availability

All data analyzed during this study are included in this published article and its supplementary information files. Specifically, the structure of all networks examined are displayed in figures or supplementary figures. GraphML representations of the full T cell, EMT, and TRRUST2 networks are provided in the repository snapshot (Additional file 5). The HiLoop repository is also available online at https://github.com/BenNordick/HiLoop.

## Abbreviations

EMT: Epithelial-mesenchymal transition
JSON: JavaScript object notation
MISA: Mutual inhibition self-activation
MISSA: Mutual inhibition single-self-activation
ODE: Ordinary differential equation
PFL: Positive feedback loop
SCC: Strongly connected component
