# Supplementary figures and images for: Identification, visualization, statistical analysis and mathematical modeling of high-feedback loops in gene regulatory networks
Source: BMC Bioinformatics. 2021 Oct 4;22:481. doi: 10.1186/s12859-021-04405-z (PMC8489061; doi:10.1186/s12859-021-04405-z)

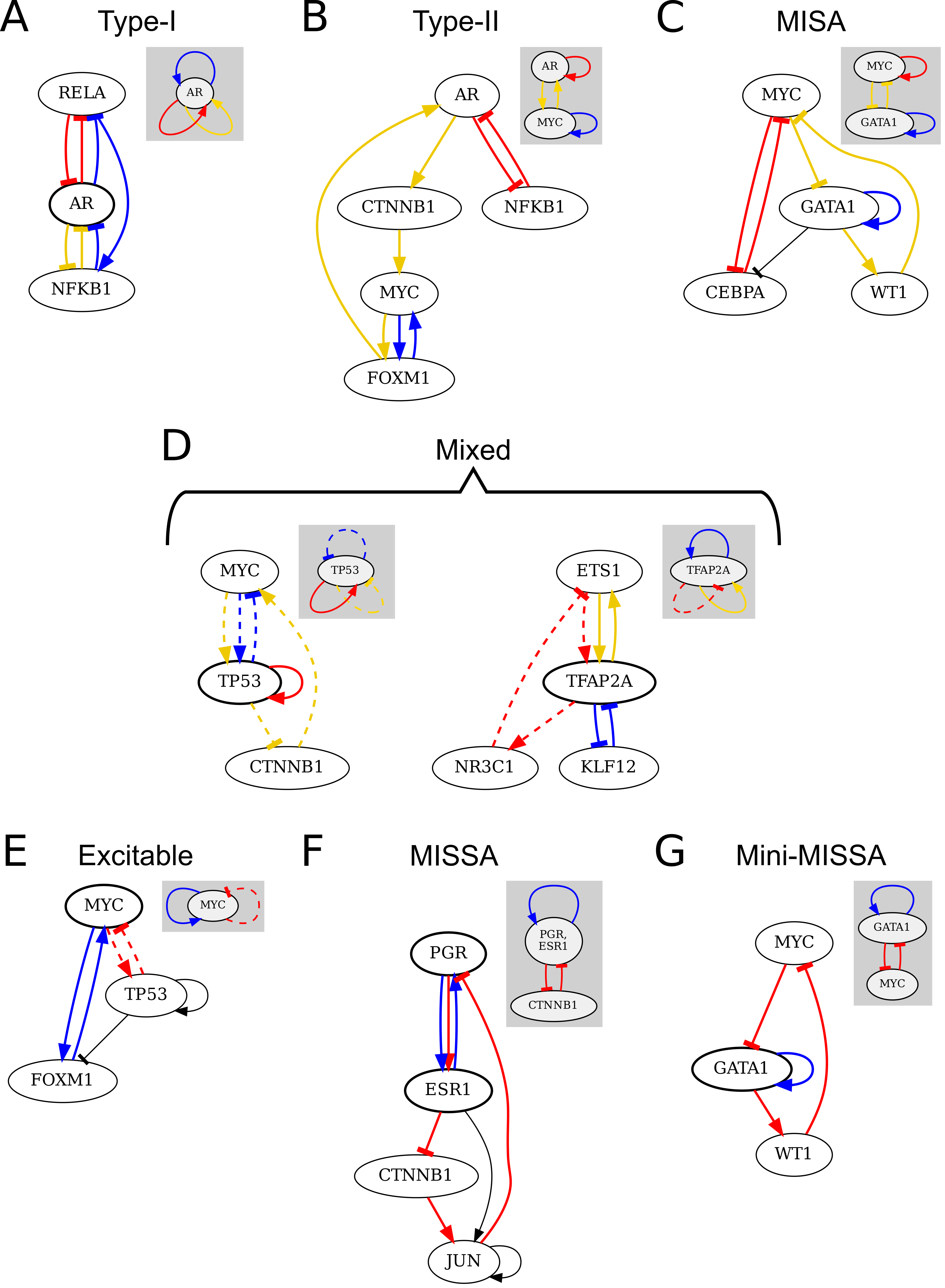

Supplement: Supplementary file 1 — Additional file 1. Figure S1: Examples of supported motifs, with logo, found in the TRRUST2 network. a A Type-I topology, consisting of three fused positive feedback loops. b A Type-II topology, consisting of two independent positive feedback loops bridged by a third net-positive loop. The bridge loop may or may not contribute mutual inhibition. c A mutual inhibition self-activation (MISA) topology, i.e. a Type-II topology with mutual inhibition between members of the two independent positive feedback loops. d Mixed-sign high-feedback topologies, consisting of three fused feedback loops, at least one of which is positive and at least one of which is negative. Dashed lines in the network diagram and logo indicate which cycles are negative. e An excitable topology, consisting of a positive feedback loop fused to a negative feedback loop. f A mutual-inhibition single-self-activation (MISSA) topology, in which a pure-activation feedback loop is fused to another positive feedback loop that does contain repressions. The self-activation may or may not be a self-loop. g A “miniature” MISSA topology in which the self-activation is a self-loop. [file 12859_2021_4405_MOESM1_ESM.png]
